# Impact of legislation and public funding on oncofertility: a survey of Canadian, French and Moroccan pediatric hematologists/oncologists

**DOI:** 10.1186/s12910-020-00466-6

**Published:** 2020-04-03

**Authors:** Aliya Oulaya Affdal, Michael Grynberg, Laila Hessissen, Vardit Ravitsky

**Affiliations:** 1 Bioethics Program, Department of Social and Preventive Medicine, School of Public Health, University of Montreal, Montréal, Québec Canada; 2 Centre de Recherche en Santé Publique, Montreal, Québec Canada; 3 Department of Reproductive Medicine & Fertility Preservation, Hôpital Antoine Beclere, Clamart, France; Université Paris Saclay, Clamart, France; 4 Pediatric Hematology and Oncology Center, Mohamed V University, Rabat, Morocco

## Abstract

**Background:**

Chemotherapy and/or radiotherapy treatments may cause premature ovarian failure and irreversible loss of fertility. In the context of childhood cancers, it is now acknowledged that possible negative effects of therapies on future reproductive autonomy are a major concern. While a few options are open to post-pubertal patients, the only immediate option currently open to pre-pubertal girls is cryopreservation of ovarian tissue and subsequent transplantation. The aim of the study was to address a current gap in knowledge regarding the offer of fertility preservation by Ovarian Tissue Cryopreservation (OTC) for prepubescent girls with cancer, and to explore current practices and attitudes of Canadian, French and Moroccan pediatric heme oncologists. The comparative perspective is relevant since legal frameworks surrounding fertility preservation and funding offered by the healthcare system vary greatly.

**Methods:**

An online survey was sent to the 45 pediatric oncology centers in Canada, France and Morocco.

**Results:**

A total of 39 centers responded (86.6%). OTC is offered by almost all pediatric heme oncologists in France (98%), very few in Canada (5%), and none in Morocco (0%). For pediatric hematologists/oncologists who do not propose fertility preservation in Canada, the reasons are: the technique is still experimental (54%), it is not available locally (26%) and cost of the technique for the family (14%). 97% of Canadian and 100% of Moroccan pediatric hematologists/oncologists think OTC should be funded by the healthcare system as it is in France and in the province of Quebec in Canada.

**Conclusions:**

The results of this study show tremendous diversity in the provision of OTC across countries, whereby its offer is correlated with legislation and funding. We argue that the current reality, in which this technology is often not offered to families, raises ethical issues related to justice and equity of access, as well as informed consent and future reproductive autonomy.

## Background

Chemotherapy and radiotherapy treatments have increased the life expectancy of cancer patients. However, depending on their aggressiveness, these treatments may cause premature ovarian failure and irreversible loss of fertility [1]. In the context of childhood cancers, it is now acknowledged that possible negative effects of therapies on future reproductive autonomy are a major concern [2, 3]. While a few options are available to *post*-*pubertal* patients (such as oocyte or embryo cryopreservation), the only immediate alternative for *pre-pubertal* girls is ovarian tissue cryopreservation (OTC) with subsequent transplantation, a procedure that is still experimental [4, 5]. The American Society of Clinical Oncology and National Comprehensive Cancer Network guidelines state that OTC is an investigational method of fertility preservation and the possibility of reseeding cancer through transplanted tissue exists [6–8]. Nevertheless, it already resulted in the birth of over 130 babies, 2 of them born from ovarian tissue that has been cryopreserved in childhood [9–11].

In 2013 and 2018, the American Society of Clinical Oncology (ASCO) recommended that patients be fully informed about the risk of infertility and fertility preservation options prior to cancer treatment [12] but it remains unclear how this guidance should be implemented in practice. Several studies concerning practices of pediatric hematologists/oncologists regarding fertility preservation in pre-pubertal girls have been recently published regarding the US [13, 14] and some European countries [15–19], but to our knowledge none is available regarding Canada, France and Morocco.

Our study aimed to address this gap and explore health professionals’ current practices and attitudes regarding OTC in these countries. The comparative perspective is relevant since legal frameworks surrounding fertility preservation and funding offered by the healthcare system vary greatly. In France, the 2004 Bioethics Law (august 6 - Article L.2141–11) requires offering fertility preservation for cancer patients while in Canada and Morocco there is no such legal recommendation. France offers funding by the healthcare system for fertility preservation for cancer patients, while in Canada (except in the province of Quebec) and Morocco no such funding exists.

## Methods

The survey was conducted in three countries: Canada, France and Morocco. Forty-five centers were identified through the “C17 Council, Canadian Centres Battling Cancer and Blood Disorders in Children” in Canada, the “French National Cancer Institute” in France and the “Moroccan Society of Pediatric Hematology and Oncology” in Morocco. A standardized email introducing the aims of the study was sent to a total of 260 pediatric hematologists/oncologists with a link to an internet- based English and French survey. Contact data was obtained from the centers’ websites.

The survey instrument (Additional file 1) was developed based on a review of the literature and designed to be brief and easy to read, so that physicians would be able to complete it in less than 10 min. The survey included questions on demographic characteristics, questions regarding knowledge of OTC, questions on who is in charge of providing counseling and on public funding by the healthcare system. Open questions were included to seek views regarding the reasons of not providing fertility preservation and the ethical issues associated with OTC. The survey also provided a free text space for participants to add any comments on the topic.

The survey was piloted on two pediatric hematologists/oncologists and two researchers to ensure readability. To increase the return rate, participants were reminded of the survey up to three times. Data was collected between February 2016 and June 2016.

Descriptive statistics were conducted to facilitate statistical analysis. Answers to questions were coded thanks to numerical values. Survey responses were exported to and graphics generated with Microsoft Excel software and SPSS for Mac version 25. Participants’ answers are presented as percentages. Qualitative data was exported to the software Nvivo to be examined and coded using thematic content analysis.

We received ethics approval for the study from the institutional review board of the University of Montréal (CÉRES) in Canada (#15–131-CERES-D). The Ethics Committee of Ibn Sina University Hospital Rabat Centre in Morocco and the Ethics Committee “CPP IDF IV” at the hospital Saint Louis in Paris waived the necessity for ethical approval because the research was not a biomedical research related to health or medical issues and an ethical approval was already obtained in Canada.

All potential participants in this study were sent an email explaining the context, objectives, methodology and expected outcomes of the study, as well as the scientific utility of their participation and a link to the survey. By clicking on the link, a “Procedure for free and informed consent” was presented with full information about the research team, study objectives and protocol. They were also informed about possible benefits of the study, as well as their right to decline the invitation and to withdraw from the study at any time. No specific risks were identified. Participants were informed that returning a completed questionnaire constituted consent to participate in the study.

## Results

Table 1 outlines the detailed demographics characteristics as reported by 96 respondents, representing almost 37% of the 260 pediatric hematologists/oncologists contacted from Canada, France and Morocco. Forty-five pediatric oncology centers were contacted and 39 responded, representing 86,6%.

**Table 1:** Demographics of participants

Demographics	Participants *N* (%)	Centers *N* (%)
Total contacted	260	45
Responses received	96 (36.9)	39 (86.6)
Canada	35 (13.5)	12 (26.7)
Ontario	11	
British Columbia	7	
Quebec	4	
Alberta	3	
Manitoba	3	
Nova Scotia	3	
Newfounland	2	
Saskatchan	2	
France	46 (17.7)	24 (53.3)
Île-de-France	13	
Auvergne-Rhône-Alpes	9	
Grand Est	4	
Provence-Alpes-Côte d’Azur	4	
Pays de La Loire	3	
Région d’outre mer	3	
Haut de France	3	
Nouvelle-Aquitaine	2	
Occitanie	2	
Bretagne	1	
Centre-Val de Loire	1	
Normandie	1	
Morocco	10 (3.8)	3 (6.6)
Rabat-Salé-Kénitra	6	
Casablanca-Settat	3	
Marrakech-Safi	1	
Not completed or not displayed	5 (1.9)	6 (13.3)
Gender		
Female	54 (56.3)	
Male	37 (38.5)	
Not completed or not displayed	5 (5.2)	
Years of practice in pediatric oncology unit		
> 1	0 (0.0)	
1–5	15 (15.6)	
5–10	16 (16.7)	
10 <	60 (62.5)	
Not completed	5 (5.2)	

**Fig. 1:**
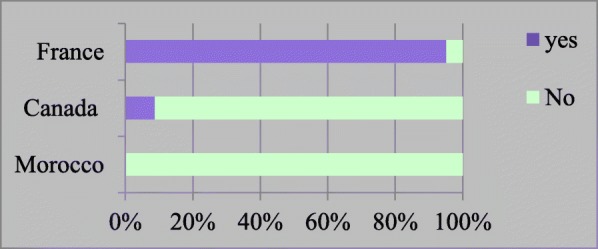
Do you offer OTC to prepubescent girls?

The results of our study show significant discrepancy in the provision of fertility preservation through OTC across countries. In France, almost all pediatric hematologists/oncologists (98%) propose OTC, while very few in Canada (5%) and none in Morocco (0%) **(**Fig. 1). In France, 2 participants from 2 different regions declared not offering OTC. In Canada, 3 participants from 2 different provinces declared offering OTC to their patients.

**Fig. 2:**
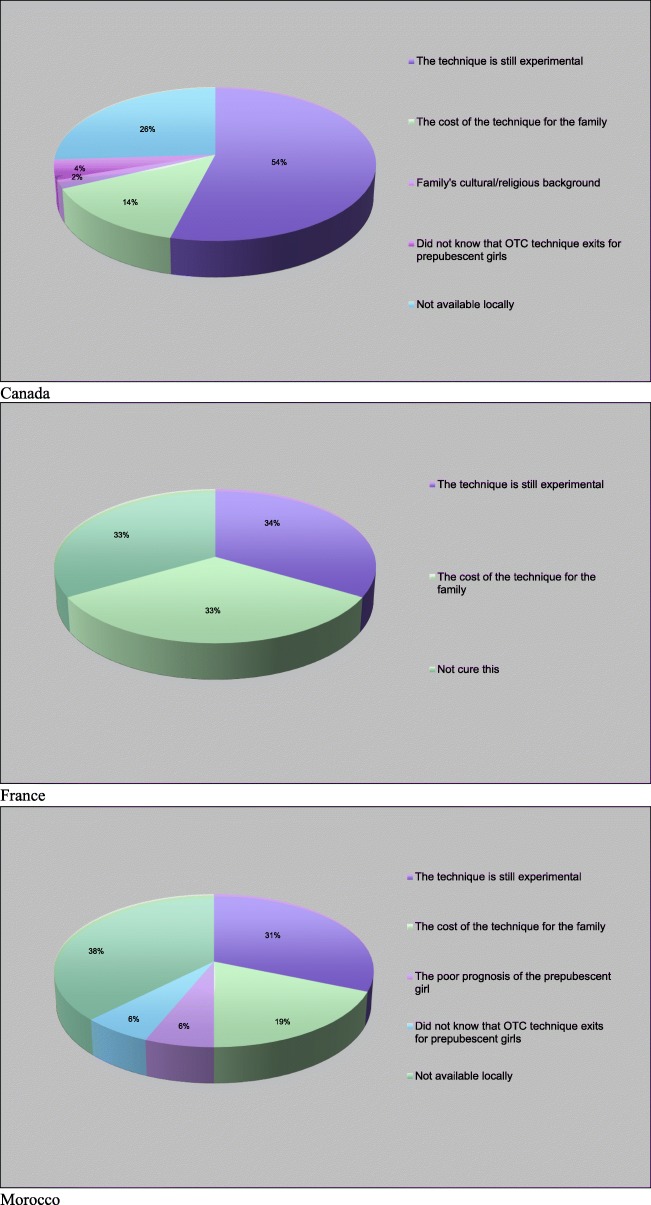
Generally, why are you not offering fertility preservation for prepubescent girls?

For pediatric hematologists/oncologists who do not propose fertility preservation, the reasons vary depending on countries. However, two main reasons remain: the technique is still experimental and the cost of the technique for the family (Fig. 2).

**Fig. 3:**
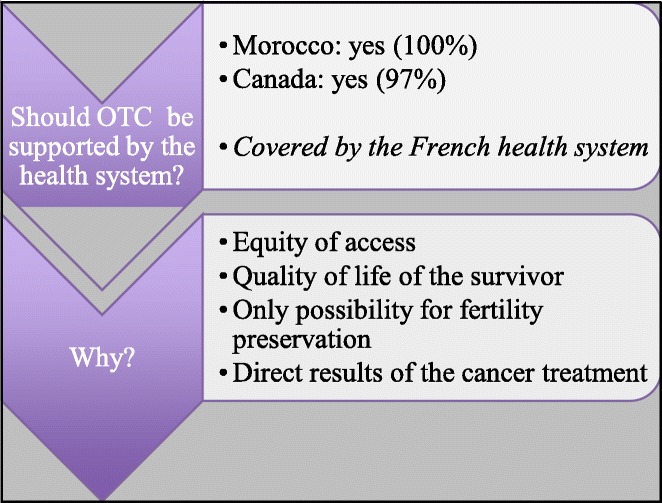
Fertility preservation for prepubescent girls is covered by France and Quebec’s public healthcare system. Do you think that it should be covered by the local healthcare system?

Additionally, 97% of Canadian and 100% of Moroccan pediatric hematologists/oncologists think that fertility preservation should be funded by the healthcare system to promote equity of access and the quality of life of cancer survivors (Fig. 3).

**Fig. 4:**
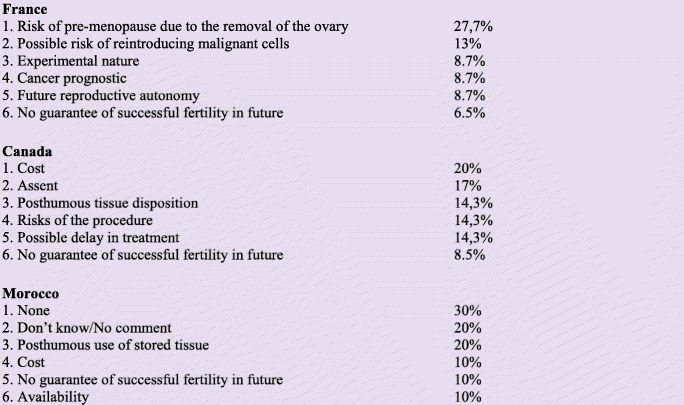
In your opinion, what are the most important ethical issues of OTC related to fertility preservation in prepubescent girls with cancer?

Cost is indeed an important reason for not offering fertility preservation. For Canadian participants in this study, cost is one of the most important ethical issue raised by OTC. It is cited by only 10% of Moroccan participants while for French participants the cost is not an ethical issue (Fig. 4).

## Discussion

In France, the offer of fertility preservation is required by a 2004 Bioethics law [20]. Systematic access to fertility preservation is also underlined by the French National Cancer Plan 2014–2019 [21], which instructs healthcare professionals to inform patients about the fertility risks of cancer treatment and offer them options. For minors, consent should be obtained from parents and the child should be as involved as possible. In contrast to France, in Canada and Morocco there is no legal recommendation to offer fertility preservation (Table 2). These results show that if we consider such offer to be ethically desirable or even required, legislation seems to be an effective method of attaining this objective.

**Table 2:** Legal framework and public funding for fertility preservation for cancer patients

	Legal framework	Public healthcare system
France	**Legal recommendation to offer** ***Bioethics Law 2004 august 6 - Article L.2141–11*** “For the subsequent realization of an Assisted Reproductive Technology, any person can benefit from the collection and preservation of his gametes or germinal tissue, when a medical treatment is likely to alter fertility or when fertility is likely to be prematurely altered.”	**Coverage by healthcare system for treatments inducing a loss of fertility** ***Social Security Code - Article D322–1*** “The list of conditions involving prolonged treatment and particularly expensive treatment that may give entitlement to withdrawal of the participation of the insured persons (...): malignant tumor, malignant disease of the lymphatic or hematopoietic tissue.”
Canada	**No legal recommendation to offer**	**No funding by healthcare system, except in the province of Quebec, where the*Law 20 Division XII.2*says:** “If rendered to a fertile insured person before any oncological chemotherapy treatment or radiotherapy treatment involving a serious risk of (…) permanent infertility, (…) the fertility preservation services listed below must be considered insured services (…): (*a*) the services required for ovarian stimulation or ovulation induction; (*b*) the services required to retrieve eggs or ovarian tissue; (…)”
Morocco	**No legal recommendation to offer**	**No funding by healthcare system**

In general, it appears that the offer of OTC for pediatric cancer patients is proportional to the funding. Thus in France, where it is covered by the healthcare system, it is widely offered. Note that in France, this coverage includes the procedure of retrieving ovarian tissue, transplant later in life and IVF, but excludes annual freezing fees [22]. In Morocco, on the other hand, there is no funding by the healthcare system and indeed, none of the Moroccan participants offer OTC (Fig. 1).

In Canada, there is inequality in terms of funding. In Quebec (a Canadian Province in which about 22% of the Canadian population lives), coverage includes the procedure of retrieving ovarian tissue and freezing fees for 5 years [23] and other provinces offers no funding. Although funding is only available in Quebec, none of the participants from this province offer OTC, which is a surprising finding. Meanwhile, 3 participants from 2 other provinces, where OTC is *not* covered, do offer it. It is therefore interesting to observe that in Canada, funding does not correlate with offer. This result could be explained by the fact that in Quebec, unlike in France, there is no legal requirement to offer fertility preservation (Table 2). Our findings show that to effectively promote a systematic offer of OTC, both legal requirement *and* funding are needed.

The funding of fertility preservation for young girls by the French healthcare system - in contrast to Canada (high income) and Morocco (lower middle income) - could be explained based on the importance of the principle of vulnerability in European bioethics. Although national health systems and funding policies vary greatly across Europe, some bioethical principles - such as “vulnerability” - are shared by European countries. Some authors have argued that bioethics in Europe has a distinct identity where, contrary to North American bioethics, the principle of vulnerability is paramount [24, 25]. In North America, four principles of biomedical ethics are widely applied and taught in medical schools: autonomy, beneficience, non-maleficience and justice [26], while in Europe, principles of vulnerability, autonomy, dignity and integrity prevail [24, 27] (Table 3).

**Table 3:** Principles of bioethics

North American Context	European Context
Autonomy	**Vulnerability**
Beneficience	Autonomy
Non-maleficience	Dignity
Justice	Integrity

The European bioethical principle of vulnerability highlights the “protection of the private sphere of humans beings” [24] and underlines the responsibility of society to protect the most vulnerable persons, such as children. Indeed, children are particularly vulnerable at the time of a life-threatening diagnosis as in the context of oncofertility. This responsibility is highlighted by French respondents who consider the risks associated with the procedure of OTC (risk of pre-menopause due to the removal of the ovary (27.7%) and possible risk of reintroducing malignant cells (13%) or experimental nature (8.7%)) as important ethical issues (Fig. 4).

Most participants do not offer OTC as an option because the technique is experimental (Canada, 54%; France, 34%; Morocco, 31%) (Fig. 2). OTC involves extracting and freezing ovarian tissue containing primordial follicles by laparoscopy before starting oncological treatment [4, 8]. Since the first OTC performed in 1999 [28], the technique has been increasingly established [29]. In 2012 and 2013, the transplantation of ovarian tissue induced puberty in a 13-year-old girl without the need of hormone treatment [30] and in a 9-year-old girl [31]. This is an important achievement since puberty can be at some points restored naturally without any medical treatment. In 2015 and 2016, this technique resulted in the birth of two children born from ovarian tissue that has been cryopreserved in childhood [9, 10] and overall, to date, it resulted in the birth of over 130 babies in women [11].

While it presents certain risks, such as reintroducing malignant cells into the body, which could theoretically propagate cancer recurrence, no study has yet shown such recurrence in humans [29]. Data is also limited regarding livebirth following chemotherapy and OTC without autotransplantation. Indeed, some women might not be sterilized following chemotherapy [32]. Furthermore, as emphasized by all participants (independently of countries), the absence of guarantee of successful fertility is an important ethical issue **(**Fig. 4**)**. Proof of efficacity in a method that might restore fertility long after treatment proves difficult and requires long-term follow-up with young prepubescent patients, introducing a possible delay of over 15 years to establish the degree of efficacy.

Current absence of robust data regarding efficacy could be used to justify denial of reimbursement by health insurances. However, emerging data underlines the importance of further research to pave the way for the offer of OTC to become the standard of care [33]. Even though OTC is still experimental -in all ages- it has the potential to become an established fertility preservation method in the near future [8, 34]. International recommendations from a 2016 expert meeting conclude that the best candidates for OTC are prepubescent girls [35]. As for any emerging technology whose effectiveness is in the process of being established, acceptance by professional societies as no longer experimental is a key step towards increasing its offer and implementation. Another element is analysis of cost-effectiveness to determine a policy approach regarding systematic offer and public funding.

In light of the findings of this study, we argue that such assessments of OTC are urgent, since not offering it could be ethically problematic from at least four perspectives: future reproductive autonomy, equity of access, vulnerability and consent.

1. **Future reproductive autonomy**: Some participants have pointed out future reproductive autonomy as an important ethical issue (Fig. 4). Barriers to fertility preservation possibly limit the future capacity to have genetically-related children, thereby limiting future reproductive autonomy and the “right to an open future” [36]. While having genetically-related children could be important to women regardless of their culture [37], the meaning of infertility varies across cultures and could present particular challenges in cultures where it can lead to stigmatization, such as in a Muslim country like Morocco [38]. This requires clinicians to take cultural values into account when respecting families’ autonomous decision-making.
2. **Equity of access**: The cost of fertility preservation is very high, ranging from $ 5000 to $ 30,000, depending on what is covered (retrieval of ovarian tissue, annual cryopreservation fees, future transplantation, and future IVF) [39]. This creates a barrier to access for many young girls and raises justice and equity issues. Participants in our study noted cost as an important reason for not offering fertility preservation (Fig. 4). This result corroborates the findings of Campbell’s study, which underlines the cost of fertility preservation as the most commonly reported barrier for pediatric oncology providers [40]. The issue of access can be resolved or alleviated by public funding, as in case of France and other European countries [41]. Cost-effectiveness analysis of OTC is therefore needed to inform policy decisions regarding public funding in countries that do not offer it.
3. **Vulnerability**: The stress caused by barriers to access may exacerbate the vulnerability of young girls and their parents at a time when they are coping with a life-threatening diagnosis and impending cancer treatment [42]. This vulnerability could also impact childhood cancer survivors when they have reached adulthood in case of infertility [43] knowing that they could have benefited from an already existing treatment.
4. **Consent and assent**: Offering OTC to all patients without financial constraints would be an important step forward, but ethical challenges also include how to present relevant information about OTC to families, considering the experimental nature of the technique and the young age of the patient. Indeed, Canadian participants highlighted consent/assent as an important ethical issue (17%). There is therefore a need to develop guidelines for clinicians and provide them with resources that facilitate these discussions with families [44].

### Towards an ethical framework for offering OTC to prepubescent girls

As highlighted by French participants, respect for future reproductive autonomy is an important ethical issue. However, practices vary considerably worldwide, depending on laws, policies, and cultural values. We argue that there is an ethical obligation to offer counselling and to discuss OTC with families of prepubescent girls in order to promote their autonomous decision-making and to allow them to consider the best interest of their daughter based on their own values.

Evaluating the best interest of a child may be a challenging task, particularly in this context, since the girl’s “best interest” covers both her *present* interest in minimizing the risk and her *future* interest in promoting reproductive choice. Clinicians have an ethical obligation to inform the patient and her parents about infertility risks and fertility preservation options. Providing information will allow them to determine the child’s best interest regarding fertility preservation. In the bioethical literature, a child’s right to fertility preservation is recognized as a *right in trust* that should be protected until he or she reaches adulthood and is capable of deciding. This ‘right to an open future’ [45] is relevant as it interprets the best interest through the lens of the child’s possible future interest in becoming a genetic parent.

Fifty percent of Moroccan participants did not recognize any ethical issue. This result underlines the lack of knowledge and awareness and the need to develop oncofertility education in Morocco. As observed by Overbeek et al. [19], lack of awareness is an important barrier, particularly in developing countries [46]. To address this issue, one option could be the establishment of centers with appropriate expertise. Indeed, to be ethically acceptable, OTC should be offered only by centers with appropriate competence to minimize the risks [35]. With time, the offer of OTC could expand to facilitate access, which requires building local expertise in the use of the technique. According to guidelines issued by the ASCO, OTC should be offered exclusively in a research setting subject to institutional review board approval [8], since benefit to the patient is not yet established [47].

Finally, guidelines for best practices in counseling are critical to an ethical offer of OTC. Discussion with a fertility specialist should ensure all relevant information is disclosed to promote informed consent and assent [44, 48]. Required general anesthesia, minor surgical procedure, need for research participation, experimental nature, and uncertainty of long-term efficacy should be discussed. An institutional program would facilitate this requirements and collaboration between the different professionals to achieve a best process of fertility preservation. Over the past few years, several programs were developed to improve fertility preservation care that had positive effects in patients [49–51]. The development of such programs could promote oncofertility services in countries where offer is currently insufficient, such as Morocco and Canada [46].

Another interesting result relates to posthumous disposition of preserved ovarian tissue. 14,3% of Canadian and 20% of Moroccan participants who do not offer OTC mentioned the disposition of stored tissue as an important ethical issue. In contrast, none of the French participants recognized this as an ethical issue. Indeed, in the tragic event of death, this disposition can be an exceptionally delicate matter. Considering the young age of prepubescent girls at the time that tissue is harvested and cryopreserved, it is impossible for some of them to give their assent for a complex matter such as disposition, whether for research, donation or – in particular – posthumous reproductive use. The difference between the responses of Canadian and French participants could be explained by the different regulatory frameworks governing posthumous reproduction in these countries. In France, posthumous reproductive use of gametes is prohibited by law, with or without prior consent. Consequently, French providers do not see it as a concern. In Canada, written consent could be sufficient to use gametes posthumously, which could explain the recognition of providers that it could be an ethically complex and sensitive issue in the case of OTC for young girls [52]. In Morocco, posthumous insemination is prohibited [53]. Regardless of the legal framework, the dispositional of preserved ovarian tissue should be addressed in all countries during the consent process at the time of procurement and preferences should be recorded for the future.

Fertility preservation through OTC is an experimental procedure – as highlighted by a majority of the participants (Fig. 2) - and it is not a guarantee for having genetic offspring in the future [54]. Long-term risks are also not well known [55]. The future risks of reintroducing malignant cells following transplantation could be mitigated by the notion that more robust data will be available in the future, before ovarian tissue will be re-implanted. At that point, the patient will not be a minor and could make that decision for herself.

### Limitations

The main limitation of this study is that it provides only the views of pediatric hematologists/oncologists and from only three countries. It may be useful to further explore the views of patients and their families, as well as practices in additional countries. This study also focuses only on fertility preservation through OTC for young girls. It may be useful to explore via a similar methodology what is offered to pubescent girls and adult women.

Concerning the limitations of the empirical methodology, the use of survey implies a result at one specific point in time and the impossibility for participants to have further information about questions when needed, this could lead to some missing data.

Finally, another limitation of our study is related to the conceptual complexity of linking normative analysis with empirical findings. While the use of empirical methods, mostly from the social sciences, has considerably increased in bioethics [56, 57] there is heated debate on the appropriate ways to link this type of research with the normative work most bioethicists engage in [57–59]. “Empirical research attempts to describe the social world as it is, while normative research seeks to describe how the world ought to be.” [60]. Clearly, empirical research does not “generate normativity” [61]. However, normative conclusions may be supported by empirical data [62]. In our study in particular, the findings shed light on the attitudes and experiences of pediatric hematologists/oncologists and offer a starting point for an ethical discussion that leads to some normative conclusions. These conclusions may contribute some insight into recommendations for future policy in the area of oncofertility. Indeed, since normatively patients and parents have a right to know their options, and empirically our survey shows many pediatric hematologists/oncologists do not offer or discuss fertility preservation, we need to promote the offer to meet normative requirements.

## Conclusions

The results of this study show tremendous diversity in the provision of OTC across France, Canada and Morocco and show that its offer is correlated with legislation and funding. The current reality, in which this technology is often not offered to and not discussed with families, raises ethical issues related to justice and equity of access, as well as informed consent and respect for future reproductive autonomy and the child’s right to an open future. Clinical guidelines should support and promote the offer and discussion of fertility preservation with children and parents. Moreover, this study shows that legal frameworks and public funding can be effective in implementing oncofertility programs.

Our findings call for further empirical studies, such as in-depth interviews to explore the needs and concerns of young girls, their families, as well as those of health professionals. Cost-effectiveness analysis is required to inform policy decisions about public funding of OTC to facilitate the implementation of the technique and reduce inequity of access. Further ethical analysis is required to consider the implications of this revolutionary technology for reproductive rights and future reproductive autonomy.

## Supplementary information

**Additional file 1.**

## Data Availability

The datasets used and/or analysed during the current study are available from the corresponding author on reasonable request.

## Abbreviation

OTC: Ovarian Tissue Cryopreservation
