# Supplementary material for: Impact of legislation and public funding on oncofertility: a survey of Canadian, French and Moroccan pediatric hematologists/oncologists
Source: BMC Med Ethics. 2020 Apr 3;21:25. doi: 10.1186/s12910-020-00466-6 (PMC7118810; doi:10.1186/s12910-020-00466-6)
Supplement: Supplementary file 1 — Additional file 1. [file 12910_2020_466_MOESM1_ESM.docx]

**Oncofertility Survey**

*Demographics*

1. Graduation date (year): ______

2. Years of practice :

6 months or less  6 months -1 year  1-5 years  5-10 years  10 years or more

3. Years of practice in the pediatric oncology unit:

6 months or less  6 months -1 year  1-5 years  5-10 years  10 years or more

4. Country: ______ 5. Province/City: ______

6. Gender:  Female  Male

**1**. Have you ever heard of Ovarian Tissue Cryopreservation (OTC)?

Yes How?  Colleagues

Scientific journals

Conferences

Other: ______________________

No

**2**. Do you offer OTC to prepubescent girls?

Yes How old was the youngest? ______________________

How many patients have you offered OTC to?________

For how long have you been offering OTC? _____________

No – ***Please go to question 4***

**3**. When you offer OTC, who is in charge of providing counseling?

You (as haematologist/oncologist)

A fertility specialist

A team

Others: ______________________

**4**. Generally, why are you not offering fertility preservation for prepubescent girls?

The technique is still experimental

The poor prognosis of the prepubescent girl

Family’s cultural/religious background

The cost of the technique for the family

I didn’t know that the OTC technique existed for prepubescent girls

Others: ______________________

**5.** Are you willing to offer it in the future?

Yes Why? ______________________

No Why? ______________________

**6**. Are there any guidelines in your institution regarding OTC?

Yes Which ones?  Clinical guidelines

Policy guidelines

Others: ______________________

No

**7**. (Question for **Canadian respondants only:**)

Fertility preservation for prepubescent girls is covered by Quebec’s public healthcare system. Do you think that it should be covered in the other provinces across Canada?

Yes Why? ______________________

No Why? ______________________

**8**. (Question for **Moroccan respondants only:**)

Fertility preservation for prepubescent girls is covered by French and Quebec’s public healthcare system. Do you think that it should be covered in Morocco?

Yes Why? ______________________

No Why? ______________________

**9**. Do you know if OTC is being offered elsewhere within your country to prepubescent girls?

Yes  Public practice _______  Private practice___________

No

**10**. Do you know if OTC is being offered elsewhere outside your country to prepubescent girls?

Yes In which country? _______

No

**11**. In your opinion, what are the most ethical issues of OTC related to fertility preservation in prepubescent girls with cancer?

______________________

_____________________

______________________

_____________________

12. Do you offer OTC for postpubescent female patients?

Yes How old is the youngest?

No

13. Do you offer OTC for prepubescent boys?

Yes How old is the youngest?

No

*If you have any additional comments, please write them below.*

______________________________________________________________________________________________________________________________________________________________________________________________________________________________________________________
